# Making a Stand for Animals

**DOI:** 10.1017/awf.2023.75

**Published:** 2023-07-14

**Authors:** Peter Sandøe

**Affiliations:** University of Copenhagen, Department of Veterinary and Animal Sciences & Department of Food and Resource Economics, Copenhagen, Denmark

*Making a Stand for Animals* is Oscar Horta’s own English translation of his 2017 book *Un Paso Adelante en Defensa de los Animales.* In the book Horta describes himself as “an animal activist and moral philosopher”, and clearly both roles have played a part in the writing of the book. Horta the activist aims to encourage, and indeed provoke, the reader – who is addressed throughout in the second person as “you” – to take up the concerns he describes, helping to bring about a radical change in the way non-human sentient animals (which I shall refer to simply as “animals”) are treated by us. Horta the moral philosopher, on the other hand, presents philosophical arguments supported by more than 40 thought experiments to try to underpin the need for change.

A strong feature about the book, and something that distinguishes it from most others in the field, is that it is accessible to readers without expertise in philosophy. The main text is free of technical jargon. References appear in extensive sets of endnotes, placed at the end of each chapter, which can be skipped by readers who just want to proceed with the argument.

In his introduction Horta sets out his two main aims. These are, first, to present the powerful reasons why we must challenge widespread human lack of concern about animals, and second, to bring about large-scale behavioural change in the ways in which animals are treated. According to Horta the latter is by far the most difficult of the two tasks, and therefore more than half of the book is dedicated to it.

Seven substantive chapters follow. In the first, Horta presents what he sees as the idea underlying all forms of bad treatment of animals. This idea he describes as “speciesism”, a term coined by the activist Richard Ryder in 1970 and subsequently popularised by the philosopher Peter Singer. He defines speciesism as “discrimination against those who don’t belong to a certain species.” Most simply, this takes the form of discrimination by humans against animals as such, but as commonly it involves discriminatory human practices privileging animals of certain species over others – e.g. favouring dogs as against pigs or rats.

The main argument Horta presents against speciesism is known as “the argument from marginal cases.” In short, since it is always wrong to discriminate against other humans, whoever they are, to defend our differential treatment of animals we need to come up with a relevant difference between humans and animals. However, all of the plausible candidates here, such as rationality, language use etc, are such that there are humans (the marginal cases) that do not possess them. Therefore, our differential treatment of animals cannot be defended.

In Chapter 2, Horta focuses on the central, morally relevant property shared by humans and animals: both groups are sentient, which means that they can feel, and thus suffer. The main task for Horta is to prevent suffering. Strangely enough, he doesn’t really discuss the other side of the coin, which is to promote positive experiences. This means he doesn’t tackle the dilemmas that arise when efforts to enable positive experiences risk causing suffering (e.g. when cats are allowed to roam while also causing suffering to other animals or risking accidents themselves).

Horta also believes that the human killing of animals is wrong. He argues that killing denies animals future positive experiences, and that since humans typically care about this, when it comes to humans, they should also care about it when it comes to animals. Finally, Horta tries to delineate the group of animals which are sentient. This, he argues, includes all vertebrates and many invertebrates. Although it is difficult to draw a precise boundary based on the level of development of the nervous system and the complexity of behaviour shown by an animal, Horta recommends a precautionary approach on to which, for example, most insects will fall into the sentient category.

Chapter 3 presents an overview of the ways in which animals are exploited by humans. The focus here is on the activities “most people take part in.” Two main groups of activities are discussed: animals used for entertainment and animals used in the production of food and clothing. The latter involves by far the largest number of animals. Oddly, in this chapter Horta does not discuss animals kept as companions, even though these probably vastly outnumber the animals used for entertainment. The conditions facing animals on farms, and during transport and slaughter, are painted in very dark colours indeed. The word “terrible” is used frequently. Many people familiar with the conditions in which farm animals are kept will agree with Horta that there is a lot to criticise here, but I fear that the very one-sided and exaggerated picture he presents will backfire. For example, it is stated that calves are raised in veal crates without mentioning that this form of production has been banned in the EU since 2007. Examples of pigs and cattle that die horrible deaths at slaughterhouses due to inefficient stunning are presented as if they are common, which I am sure they are not – at least, in the EU, where animal slaughter is inspected by government-appointed inspectors. The chapter also contains simple falsehoods, probably based on hearsay (e.g. on p. 57 it is stated that “castration makes animals grow faster”). I think Horta could have made a much more trustworthy and efficient case regarding the problems facing farm animals without using hyperbole and by carefully consulting the relevant veterinary and animal science literature.

In Chapter 4, Horta begins his discussion of what should be done about the problems described in the previous chapter. His key proposal is that we should adopt a vegan lifestyle, understood in a very broad sense. Veganism, according to Horta, “is not simply a way of eating” where all animal products are avoided. It involves, more generally, not “participating in practices that make animals suffer or die.” Examples of activities avoided by the vegan, as Horta understands that outlook, include attending circuses (with animals) or going hunting or fishing. However, Horta does not discuss other potential avoidances that may be more burdensome. What about the control of rats and other so-called “pest animals” which pose a genuine risk to human health and livelihood? I personally think that we may kill rats that invade our houses, but should do so in the most humane way possible, and that we should do what is possible to prevent them from getting in. However, asking us not to kill them when they *have* got into the house seems to be an extreme requirement. On this issue Horta is largely silent. (Admittedly, the issue of killing animals in self-defence is briefly mentioned in a subsequent chapter). Nor does he ask whether the vegan lifestyle would require us not to have companion dogs and cats. Here, cats are an extremely interesting example, since they are strict carnivores and need to be fed animal products to avoid health problems.

In Chapter 5, Horta delves further into the question of how we are to live in a way that avoids speciesism. Two interesting discussions from this chapter are worth mentioning here. The first asks whether it is acceptable to eat animals raised in good conditions and subsequently killed in a painless manner. Here, Horta argues at great length that such a form of production is unrealistic, but he also says that it would in any case be unacceptable. His argument is that we would never accept a use of fellow humans that involved their being killed – e.g. for the harvesting of their organs – however well the individuals were treated prior to their being killed. This leads on to the second interesting discussion, which asks whether it is acceptable to kill animals if they live good lives and *wouldn’t* have existed but for our use of them – something that may be true of some luxury beef cattle that are raised and killed painlessly on the pasture where they live. Famously, practices meeting these two conditions (good lives and painless killing) were accepted by Peter Singer, whom Horta otherwise seems largely to follow. However, Horta rejects them, citing the argument just mentioned – that most people would not accept such a practice involving human beings. Here, an obvious rejoinder is that there may be a relevant difference between humans and typical farm animal species, e.g. in their ability to make long-term plans and fear for the future, in light of which the different treatment of animals need not be deemed to be an expression of speciesism. It would have been interesting to see Horta, the philosopher, discuss this, but clearly Horta, the activist, with a clear vegan agenda, had the upper hand.

In Chapter 6, Horta addresses some issues not yet raised in previous chapters. First, he looks, very briefly, at the idea that our ownership of dogs as companion animals is not bad for the affected animals. The idea is dismissed in the following way: “… the overall situation of dogs really is terrible. Some enjoy happy lives, but they are minority. Many of them are abandoned and die, suffering a lot in the process, often not long after birth. Others are raised in puppy mills which are similar to factory farms. Others are kept in appalling conditions, tied all their life to a short rope, enjoying no company, locked down in tiny places, sometimes suffering from hunger or being beaten up every now and then” (p. 138). Interestingly, there are no references to back up these claims which, in the view of the present reviewer, who works a lot with epidemiological and questionnaire data on companion dogs in the Global North, are clearly hyperbolical. Companion dogs have many welfare problems, and many of these are serious, but to claim that only a minority live good lives is clearly an extreme exaggeration. Moreover, it would have been of great interest to see Horta, the philosopher, engaging in discussion along the following lines: it is known that a significant number of children suffer severe welfare problems, but should we for that reason recommend that humans stop having them so that humans die out thereby stopping the childhood suffering once and for all? If the answer to that question is negative, Horta’s rejection of the practice of having companion dogs is bound to seem problematic.

Another issue raised in Chapter 6 is wild animal suffering. Here, in his previous research Horta has made an important contribution: while most of those concerned about wild animal welfare focus on so-called anthropogenic effects – that is, harm caused by human activities – Horta argues that there is reason to intervene to prevent, or at any rate alleviate, the suffering of wild animals irrespective of whether it was brought about by us. His argument is that since most people think there is an obligation for people in the rich countries to fight diseases and other serious problems facing people in poor countries irrespective of whether these problems are caused by the rich countries, parallel reasoning should lead us to conclude that we have a similar obligation when it comes to wild animals. According to Horta, for example, mass vaccination of wild animals for the sake of their welfare, similar to the vaccination of foxes against rabies to protect human health and welfare already taking place, is desirable. It should be undertaken on behalf of, and for, wild animals. Denial of this would be an expression of speciesism. Following this Horta launches a head-on attack on mainstream conservation biology. For example, he sees the killing of invasive species such as ruddy ducks in Europe and feral horses in North America as indefensible. The argument is that “something is being done to animals that would never be done to humans, a clear instance of speciesism in action” (p. 147).

Towards its end, in Chapter 6 and the concluding Chapter 7, the book takes on a lofty and moralistic tone replete with them-and-us rhetoric of the kind exhibited in the following appeals: “Many people feel pushed to accept speciesist views simply because other people do too. But we shouldn’t allow ourselves to be pulled along by what the majority happens to think, as this bias leads us to do. Rather, we should think for ourselves and make our own decisions” (p. 156). “… we have the opportunity to put ourselves on the right side of history. We can do this by abandoning the attitudes resulting from the prejudices and moral myopia of our time” (p. 167). “It’s in our hands to leave as our legacy a better world for sentient beings, both for those who live now and for those who will exist in the future. There’s a lot we can do for them. We can start today.” This could turn off some readers, and it certainly turned me off. However, more importantly, it may serve to discourage some important discussions about how to make progress when it comes to our moral obligations to animals.

I imagine that many of *Animal Welfare*’s readers are animal welfare scientists. They would naturally share quite a few of Horta’s concerns about preventing the animal suffering caused directly or indirectly by human practices. However, their perspective will typically differ from Horta’s in at least two respects. First, they will expect careful documentation of the actual welfare consequences for the animals are of the various ways in which they are bred, kept and handled. Here, there is of course a risk of myopia – failure to see the big picture of human exploitation of animals. But, as indicated above, Horta’s cavalier approach suffers from the opposite problem of grandstanding and hyperbole. Second, animal welfare scientists will very probably prioritise efforts to make a difference for the vast number animals that will be around for the foreseeable future, despite Horta’s appeals for radical change.

In my view, this book would have been a much better one if Horta had risked discussing the possible, but small, steps we could take to move away from speciesism. In the world there are already strong organisations, like PETA, which share Horta’s vegan vision but still engage in efforts to reform practices in animal production in a more humane direction. Strong philosophical voices, like that of Peter Singer, also endorse piecemeal approaches to some of the challenges of our time while still upholding a radical vision not that different from Horta’s. It would have been great to see a balanced discussion of these more pragmatic approaches. Again, Horta might have dared to discuss more openly the difficult issues raised by the practice of killing animals with the aim of improving overall welfare. The likely outcome of this would, of course, have been that Horta the philosopher would not have been able to deliver the simple messages preferred by Horta the animal activist, but still the result would have been a better book, in my view (full disclosure: I am more of a philosopher than an activist).

Can I recommend Horta’s book to my colleagues working in animal welfare science? I think that for many of them reading it would be a culture shock, and that it may well confirm many of their prejudices about both philosophers and animal activists. However, if they can see through this, they should be able enjoy and learn from grappling with the many simple, yet challenging arguments, mostly based on thought experiments, that Horta presents in the book. So, my advice would be to take a deep breath; be ready to live with the absence, or at best the selective use, of scientific evidence; and mobilise a playful and open mind; and then you are likely to have a good time with this book, and to learn from it.

